# A systematic review of cooling for neuroprotection in neonates with hypoxic ischemic encephalopathy – are we there yet?

**DOI:** 10.1186/1471-2431-7-30

**Published:** 2007-09-05

**Authors:** Sven M Schulzke, Shripada Rao, Sanjay K Patole

**Affiliations:** 1 Department of Neonatal Paediatrics, Women's and Children's Health Service, Perth, Australia; 2 University of Western Australia, Perth, Australia

## Abstract

**Background:**

The objective of this study was to systematically review randomized trials assessing therapeutic hypothermia as a treatment for term neonates with hypoxic ischemic encephalopathy.

**Methods:**

The Cochrane Central Register of Controlled Trials, MEDLINE, EMBASE, CINAHL databases, reference lists of identified studies, and proceedings of the Pediatric Academic Societies were searched in July 2006. Randomized trials assessing the effect of therapeutic hypothermia by either selective head cooling or whole body cooling in term neonates were eligible for inclusion in the meta-analysis. The primary outcome was death or neurodevelopmental disability at ≥ 18 months.

**Results:**

Five trials involving 552 neonates were included in the analysis. Cooling techniques and the definition and severity of neurodevelopmental disability differed between studies. Overall, there is evidence of a significant effect of therapeutic hypothermia on the primary composite outcome of death or disability (RR: 0.78, 95% CI: 0.66, 0.92, NNT: 8, 95% CI: 5, 20) as well as on the single outcomes of mortality (RR: 0.75, 95% CI: 0.59, 0.96) and neurodevelopmental disability at 18 to 22 months (RR: 0.72, 95% CI: 0.53, 0.98). Adverse effects include benign sinus bradycardia (RR: 7.42, 95% CI: 2.52, 21.87) and thrombocytopenia (RR: 1.47, 95% CI: 1.07, 2.03, NNH: 8) without deleterious consequences.

**Conclusion:**

In general, therapeutic hypothermia seems to have a beneficial effect on the outcome of term neonates with moderate to severe hypoxic ischemic encephalopathy. Despite the methodological differences between trials, wide confidence intervals, and the lack of follow-up data beyond the second year of life, the consistency of the results is encouraging. Further research is necessary to minimize the uncertainty regarding efficacy and safety of any specific technique of cooling for any specific population.

## Background

Hypoxic ischemic encephalopathy (HIE) following perinatal asphyxia contributes significantly to neonatal mortality and morbidity including long-term neurodevelopmental sequelae in up to 25%-60% of survivors [1-3]. Despite significant research there is still no proven intervention for neuroprotection in HIE [4]. The literature on therapeutic hypothermia as a treatment for "white asphyxia" dates as far back as early 60s [5,6]. Experimental studies have shown that mild to moderate hypothermia (33–34°C), applied within the first hours of an acute hypoxic event, is neuroprotective [7-9]. A Cochrane review incorporating two small randomized controlled trials (RCT) has reported no evidence of benefit or harm related to therapeutic hypothermia in term neonates (N = 50) with HIE [4]. Three large RCTs have been published since the last substantive amendment of this systematic review in July 2003 [10-12]. Given the significance of the condition, and the encouraging results of the recent RCTs, an up to date systematic review was conducted to evaluate the efficacy and safety of therapeutic hypothermia in term neonates with HIE [13,14].

## Methods

RCTs comparing therapeutic hypothermia, by either selective head cooling or whole body cooling, with normothermia in term neonates with perinatal asphyxia and HIE were eligible. Asphyxia was considered to be present if at least one of the following criteria was met: Apgar score of ≤ 5 at 10 minutes, at least one cord pH or arterial pH ≤ 7.1 or base deficit ≥ 12 within the first hour of life, ongoing resuscitation or mechanical ventilation at 10 minutes of life. HIE had to be defined by standardized neurological examination [15,16]. Neonates with major congenital abnormalities were excluded.

The primary outcome was a composite of death or neurodevelopmental disability at ≥ 18 months of life. Disability included cerebral palsy (CP) according to the Gross Motor Function Classification System (GMF) [17] or another validated scale, developmental delay as measured by Griffiths or Bayley assessment [18,19], intellectual impairment (IQ > 2 SD below the mean), blindness (vision < 6/60 in both eyes), and hearing loss requiring amplification. Secondary outcomes included individual components of the primary composite outcome and adverse events such as sinus bradycardia, arrhythmia, arterial hypotension (mean arterial pressure < 40 mm Hg), thrombocytopenia, coagulopathy, anemia, hypoglycemia, abnormal renal function (urine output < 0.5ml/kg/hour and/or serum creatinine > 0.09 mmol/l), hepatic dysfunction (aspartate aminotransferase > 200 IU/l, alanine aminotransferase > 100 IU/l), sepsis, seizures and hypokalemia.

A systematic literature search was conducted in July 2006 according to the methodology of the Cochrane Neonatal Review Group. The databases searched included the Cochrane Database of Systematic Reviews (Issue 3, 2006), the Cochrane Central Register of Controlled Trials, EMBASE, CINAHL, and MEDLINE databases using the following search strategy: "Infant, Newborn" [MeSH] AND ("Hypothermia" [MeSH] OR "Hypothermia, Induced" [MeSH]) AND ("Asphyxia" [MeSH] OR "Asphyxia Neonatorum" [MeSH] OR "Hypoxia-Ischemia, Brain" [MeSH] OR "hypoxic ischemic encephalopathy" OR "hypoxic ischemic encephalopathy"). Cross-references of publications were checked. A hand search of the proceedings of the Pediatric Academic Societies published in *Pediatric Research *from 1980 was conducted. No language restrictions were applied. SKP and SMS designed the review protocol. All authors searched the literature independently and assessed inclusion criteria and quality of the trials. SMS and SR independently extracted the data. Inconsistencies were sorted out by discussion. Trial quality was assessed by method of randomization, concealment of patient allocation, blinding of intervention, blinding of outcome assessors, and completeness of follow-up.

Meta-analysis was performed using Review Manager software (RevMan, version 4.2.7 for Windows, Oxford, England: The Cochrane Collaboration, 2003). Relative risk (RR) and risk difference (RD) were calculated with 95% confidence intervals (CI). Preplanned subgroup analysis for selective head cooling and whole body cooling was carried out. The number needed to treat (NNT) or harm (NNH) was calculated for significant comparisons. Heterogeneity was estimated by the I^2 ^statistic. A fixed effects model was used. Reporting follows the QUOROM guidelines [20].

## Results

187 abstracts were identified. Twelve potentially relevant reports were retrieved for detailed evaluation. A total of eight reports [10-12,21-25] of five RCTs involving 552 neonates were eligible for inclusion in the analysis. Two RCTs involved selective head cooling by a cooling cap [10,21], the other three involved whole body cooling [11,12,24]. Tables 1, 2, 3 summarize their characteristics and quality assessment. No significant heterogeneity was noted using the I^2 ^statistic. Gunn *et al *published their results as 3 different reports using 4 sequential temperature ranges for cooled infants [21-23]. From these 3 reports, we present the combined data on all neonates randomized to a rectal temperature of 34.0–35.5°C and refer to the trial as Gunn *et al *1998. Eicher *et al *reported the safety [25] and efficacy [12] outcomes of therapeutic hypothermia in two separate publications. For the purpose of this meta-analysis their study is referred to as Eicher *et al *2005. The studies Eicher *et al *2005 and Shankaran *et al *2002 [24] were included in the review only for analysis of mortality and adverse events because the follow-up rate in Eicher *et al *2005 was low (68%) and Shankaran *et al *2002 did not report long-term follow up. The reasons for excluding four trials [26-29] from the meta-analysis and their characteristics are summarized in Table 4.

**Table 1:** Characteristics of included trials

**Included trials**	**Selective head cooling**		**Whole body cooling**		
	**Gunn 1998**	**Gluckman 2005**	**Shankaran 2002**	**Shankaran 2005**	**Eicher 2005**

**Design**	RCT	RCT Multicenter	RCT Multicenter	RCT Multicenter	RCT Multicenter
**Number of participants**	26	234	19	208	65
**Gestation**	≥ 37 wks	≥ 37 wks	≥ 36 wks	≥ 37 wks	≥ 35 wks
**Inclusion criteria (details see Table 2)**	Asphyxia and moderate to severe HIE	Asphyxia and moderate to severe HIE	Asphyxia and moderate to severe HIE	Asphyxia and moderate to severe HIE	Asphyxia and HIE
**Target temperature treatment group (°C)**	34.5–35.5 (n = 6) 34.0–35.0 (n = 7) Not analyzed: 36.0–36.5 (n = 6) 35.5–35.9 (n = 6)	34.0–35.0	34.5	33.5	32.5–33.5
**Target temperature control group (°C)**	36.8–37.2	36.5–37.5	36.5	36.5–37.0	36.5–37.5
**Site of temperature probe**	Rectal	Rectal	Esophageal	Esophageal	Rectal
**Average age at start of cooling (min)**	294	330	318	302	120
**Duration of cooling (h)**	48–72	72	72	72	48
**Cooling device**	Servo-controlled cooling cap	Servo-controlled cooling cap	Two servo-controlled cooling blankets	Two servo-controlled cooling blankets	Ice bags followed by one servo-controlled cooling blanket
**Primary outcome**	Adverse events	Composite of death/severe disability	Adverse events	Composite of death/moderate or severe disability	Adverse events
**Latest follow-up (months)**	18	18	Follow-up not reported	18–22	12
**Follow-up tools**	Bayley II	Bayley II GMF	-	Bayley II GMF	Bayley II Vineland

**Table 2:** Inclusion criteria of included trials

	**Selective head cooling**		**Whole body cooling**		
	**Gunn 1998**	**Gluckman 2005**	**Shankaran 2002**	**Shankaran 2005**	**Eicher 2005**

**Definition of asphyxia**	5 min Apgar < 7 or pH < 7,1 (first hour)	10 min Apgar < 6 or resuscitation or pH < 7.0 or BD^# ^> 16 mmol (first hour)	pH < 7.0 or BD > 16 or (if no blood gas or pH 7.01–7.15 or BD^# ^10–15 mmol: acute perinatal event and seizures or HIE*)	10 min Apgar < 6 or resuscitation or pH < 7.0 or BD^# ^> 16 or (if no blood gas or pH 7.01–7.15 or BD^# ^10–15 mmol: acute perinatal event and seizures or HIE*)	10 min Apgar < 6 or resuscitation or pH < 7.0 or BD^# ^> 13 or acute perinatal event
Definition of HIE*	Sarnat stage 2–3	Pre-randomisation EEG and Sarnat stage 2–3	Modified Sarnat stage 2–3	Modified Sarnat stage 2–3	Sarnat stage 1–3

^#^BD base deficit * Hypoxic ischemic encephalopathy

**Table 3:** Quality assessment of included trials

**Included Trial**	**Gunn 1998**	**Gluckman 2005**	**Shankaran 2002**	**Shankaran 2005**	**Eicher 2005**
**Adequacy of method of randomisation**	Yes Computer generated	Yes Computer generated, block	Yes Computer generated, block	Yes Computer generated, block	Yes Central website, block
**Concealment of allocation**	Yes Sealed opaque envelopes	Yes Sealed opaque envelopes	Yes Central data- coordinating center	Yes Central data- coordinating center	Yes Central website
**Blinding of intervention**	None Due to nature of intervention	None Due to nature of intervention	None Due to nature of intervention	None Due to nature of intervention	None Due to nature of intervention
**Blinding of outcome assessors**	Not robustly	Yes	No	Yes	Unknown
**Completeness of follow-up**	Yes (96%)	Yes (93%)	Follow-up not reported^#^	Yes (98%)	No (68%)^#^

^# ^Data from this trial was only pooled for analysis of mortality and adverse events

**Table 4:** Trials excluded from the analysis

**Study**	**Description of the trial**	**Reason for exclusion**
**Akisu 2003 **[26]	Randomized trial of 21 neonates with asphyxia, 10 assigned to head cooling for 72 h, 11 assigned to normothermia. Primary outcomes were electroencephalographic changes and concentrations of platelet activating factor in cerebrospinal fluid during the time of treatment	1. Enrolment based on presence of asphyxia only, not HIE* 2. Target temperature in hypothermia group 36.0–36.5°C 3. Time of start of intervention not standardized 4. No clinical follow-up data available
**Zhou 2003 **[27]	Randomized trial of 50 term neonates with asphyxia, 27 assigned to head cooling for 72 h, 23 assigned to normothermia. Primary outcomes were echocardiographic changes at the end of the intervention	1. Inclusion based on presence of asphyxia only, not HIE* 2. No clinical follow-up data available
**Shao 2005 **[28]	Multicenter randomized trial. 206 term neonates with HIE*, 127 allocated to head cooling via cooling cap for 72 h, 79 assigned to normothermia. Primary outcome was a composite of death or severe disability at 18 months	1. Concerns about randomization: Large difference in group sizes, method of randomization unknown, concealment of allocation unknown 2. Ongoing follow-up, currently 45% of survivors assessed at 18 months
**Lin 2006 **[29]	Singlecenter trial. 58 term neonates with HIE*, 30 assigned to head cooling via cooling cap for 72 h, 28 controls allocated to normothermia. Primary outcomes were changes on head computed tomography scans after one week and a behavioral assessment at 7–10 days	1. Not randomized, group allocation based on odd or even date of admission 2. Mean temperature in control group at begin of trial 35.7°C (all neonates outborn, no transport cot available), timing of enrolment and rewarming of control group unclear 3. No follow-up data available

* Hypoxic ischemic encephalopathy

### Results of the meta-analysis

#### (1) Death or neurodevelopmental disability at ≥ 18 months (Figure 1)

**Figure 1:**
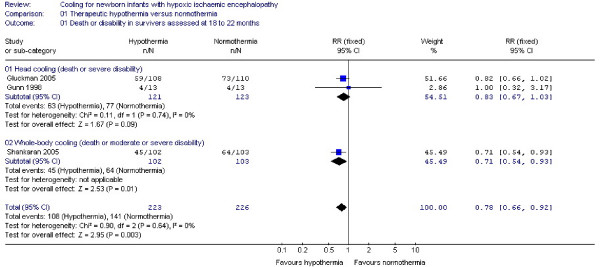
**Primary outcome: death or neurodevelopmental disability at 18 to 22 months**. Forest plot displays relative risk of death or neurodevelopmental disability at 18 to 22 months for all included studies and separately for selective head cooling and whole body cooling studies. There is a significant benefit of therapeutic hypothermia on the composite outcome of death or disability (RR: 0.78, 95% CI: 0.66, 0.92, NNT: 8, 95% CI: 5, 20). The squares represent the point estimate of treatment effect of each study with a horizontal line extending on either side of the square representing the 95% confidence interval. The diamonds represent the overall and subgroup relative risk estimate of the studies presented in the meta-analysis. The widths of the diamonds represent the 95% confidence interval of the relative risk. The vertical midline of the forest plot corresponding to a relative risk of 1 represents a "no effect" line. Gunn *et al *1998 and Gluckman *et al *2005 assessed *death or severe disability*, Shankaran *et al *2005 reported on *death or moderate or severe disability *as described in the results.

Three trials [10,11,21] involving 449 neonates assessed the primary composite outcome of death or neurodevelopmental disability at 18 to 22 months of life. The trials by Gunn *et al *1998 and Gluckman *et al *2005 (selective head cooling by a cooling cap) had primary composite outcome of *death or *s*evere neurodevelopmental disability *at 18 months defined as severe CP (equivalent to GMF level 3 to 5 in Gluckman *et al *2005), Bayley mental developmental index (MDI) < 70, or bilateral cortical visual impairment.

The trial of Shankaran *et al *2005 (whole body cooling) had a primary composite outcome as *death or moderate or severe neurodevelopmental disability *at 18 to 22 months. Moderate disability was defined as Bayley MDI of 70 to 84 in addition to one or more of the following: GMF level 2, hearing impairment with no amplification, or a persistent seizure disorder. Severe disability was defined as any of the following: GMF level 3 to 5, Bayley MDI < 70, hearing impairment requiring hearing aids, or blindness.

There is evidence of a significant effect of therapeutic hypothermia on the primary composite outcome of death or disability (RR: 0.78, 95% CI: 0.66, 0.92, NNT: 8, 95% CI: 5, 20) on pooling the data from these 3 trials. Analysis of the data from Shankaran *et al *2005 showed evidence of benefit of whole body cooling in reducing death or moderate or severe disability (RR: 0.71, 95% CI: 0.54, 0.93, NNT: 6, 95% CI: 3, 20). Analysis of data from Gunn *et al *1998 and Gluckman *et al *2005 (selective head cooling) did not indicate such an effect on death or severe disability considering the 95% CI (RR: 0.83, 95% CI: 0.67, 1.03).

The results for the primary composite outcome did not change significantly on analysis by a random effects model.

#### (2) Mortality (Figure 2)

**Figure 2:**
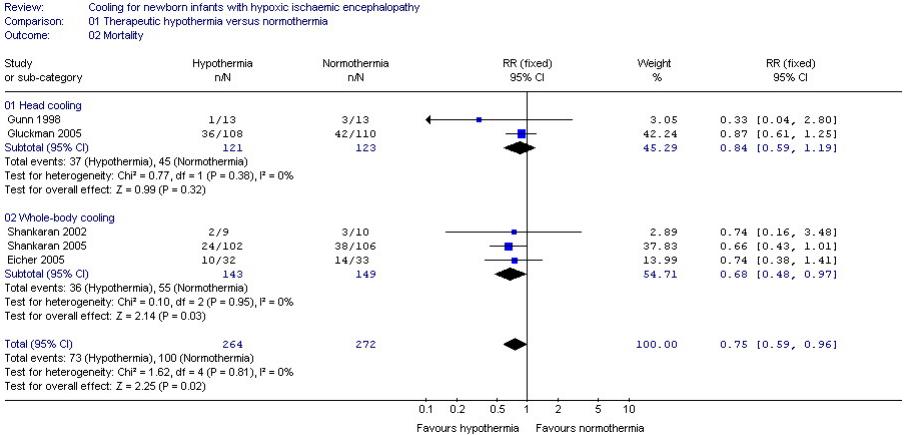
**Secondary outcome: mortality**. Forest plot displays relative risk of death for all included studies and separately for selective head cooling and whole body cooling studies. There is a significant benefit of therapeutic hypothermia on the single outcome of mortality (RR: 0.75, 95% CI: 0.59, 0.96, NNT: 11, 95% CI: 6, 100). The squares represent the point estimate of treatment effect of each study with a horizontal line extending on either side of the square representing the 95% confidence interval. The diamonds represent the overall and subgroup relative risk estimate of the studies presented in the meta-analysis. The widths of the diamonds represent the 95% confidence interval of the relative risk. The vertical midline of the forest plot corresponding to a relative risk of 1 represents a "no effect" line.

Overall, analysis of data from five trials [10-12,21,24] demonstrated a beneficial effect of therapeutic hypothermia on mortality (RR: 0.75, 95% CI: 0.59, 0.96, NNT: 11, 95% CI: 6, 100). Subgroup analysis indicated no significant reduction of mortality after selective head cooling (RR: 0.84, 95% CI: 0.59, 1.19). However, whole body cooling did have a protective effect (RR: 0.68, 95% CI: 0.48, 0.97, NNT: 8, 95% CI: 5, 100).

#### (3) Neurodevelopmental disability at ≥ 18 months (Figure 3)

**Figure 3:**
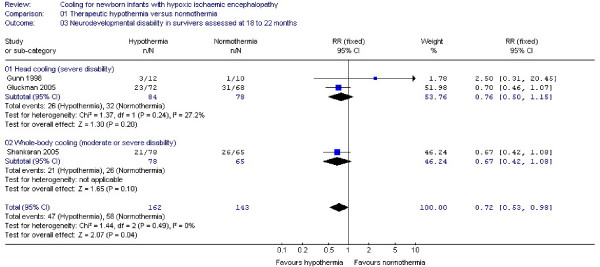
**Secondary outcome: neurodevelopmental disability at 18 to 22 months**. Forest plot displays relative risk of neurodevelopmental disability at 18 to 22 months for all included studies and separately for selective head cooling and whole body cooling studies. There is a significant benefit of therapeutic hypothermia on the single outcome of neurodevelopmental disability at 18 to 22 months (RR: 0.72, 95% CI: 0.53, 0.98, NNT: 9, 95% CI: 5, 100). The squares represent the point estimate of treatment effect of each study with a horizontal line extending on either side of the square representing the 95% confidence interval. The arrowheads indicate a wide confidence interval that is compressed to fit the scale. The diamonds represent the overall and subgroup relative risk estimate of the studies presented in the meta-analysis. The widths of the diamonds represent the 95% confidence interval of the relative risk. The vertical midline of the forest plot corresponding to a relative risk of 1 represents a "no effect" line. Gunn *et al *1998 and Gluckman *et al *2005 assessed *severe disability*, Shankaran *et al *2005 reported on *moderate or severe disability *as explained in the results.

Analysis of data from three trials [10,11,21] showed a significant effect of therapeutic hypothermia on neurodevelopmental disability at 18 to 22 months (RR: 0.72, 95% CI: 0.53, 0.98, NNT: 9, 95% CI: 5, 100). Subgroup analysis of trials of neither selective head cooling assessing *severe *disability (RR: 0.76, 95% CI: 0.50, 1.15) nor whole body cooling assessing *moderate or severe *disability (RR: 0.67, 95% CI: 0.42, 1.08) showed a significant benefit.

#### (4) Disabling CP (GMF level 3 to 5) at ≥ 18 months

Pooled data from three trials [10,11,21] didn't show a significant effect of therapeutic hypothermia on disabling CP at 18 to 22 months (RR: 0.69, 95% CI: 0.46, 1.03). Subgroup analysis of selective head cooling (RR: 0.72, 95% CI: 0.41, 1.26) or whole body cooling (RR: 0.66, 95% CI: 0.36, 1.18) also did not indicate a significant effect.

#### (5) Developmental delay (Bayley MDI < 70) at ≥ 18 months

Analysis of data from three trials [10,11,21] didn't demonstrate a significant effect of therapeutic hypothermia on developmental delay at 18 to 22 months (RR: 0.76, 95% CI: 0.54, 1.06). Subgroup analysis of selective head cooling (RR: 0.86, 95% CI: 0.54, 1.37) or whole body cooling (RR: 0.65, 95% CI: 0.40, 1.08) also did not indicate a significant benefit.

#### (6) Blindness at ≥ 18 months

Pooled data from three trials [10,11,21] didn't indicate a significant effect of therapeutic hypothermia on blindness assessed at 18 to 22 months (RR: 0.52, 95% CI: 0.27, 1.02). Subgroup analysis of selective head cooling (RR: 0.57, 95% CI: 0.23, 1.37) or whole body cooling (RR: 0.47, 95% CI: 0.16, 1.32) also did not indicate a significant benefit.

#### (7) Hearing loss requiring amplification assessed at ≥ 18 months

Analysis of data from three trials [10,11,21] didn't indicate a significant effect of therapeutic hypothermia on severe hearing loss at 18 to 22 months (RR: 0.97, 95% CI: 0.36, 2.59). Subgroup analysis of selective head cooling (RR: 1.43, 95% CI: 0.36, 5.72) or whole body cooling (RR: 0.62, 95% CI: 0.14, 2.68) also did not indicate a significant effect.

#### (8) Adverse events

Sinus bradycardia [12,15,17,30] (RR: 7.42, 95% CI: 2.52, 21.87, NNH: 13, 95% CI: 8, 20) and thrombocytopenia [12,15,17] (RR: 1.47, 95% CI: 1.07, 2.03, NNH: 8, 95% CI: 5, 50) were reported as significant adverse events (Thrombocytopenia: Gunn *et al *1998 and Eicher *et al *2005: platelet count < 150000/μl, Gluckman *et al *2005: < 100000/μl). Only Gluckman *et al *2005 reported higher mean plasma glucose concentrations between 4 h and 24 h in cooled vs control infants which resolved spontaneously [10]. There were no other significant adverse effects (Table 5).

**Table 5:** Analysis of adverse effects of therapeutic hypothermia

**Number of included trials**	**Number of participants**	**Adverse event**	**Relative risk (95% CI)**
4 [10,11,21,25]	526	Sinus bradycardia	7.42 (2.52, 21.87) Number needed to harm: 13 (95% CI: 8, 20)
3 [10,11,21]	464	Arrhythmia requiring treatment	1.04 (0.07, 16.39)
4 [10,11,21,24]	483	Hypotension	1.17 (0.96, 1.42)
3 [10,21,25]	318	Thrombocytopenia	1.47 (1.07, 2.03) Number needed to harm: 8 (95% CI: 5, 50)
3 [10,11,25]	500	Coagulopathy	1.28 (0.94, 1.75)
2 [10,25]	292	Anemia	1.75 (0.86, 3.57)
3 [10,11,21]	464	Hypoglycemia	0.76 (0.49 to 1.17)
5 [10,11,21,24,25]	545	Abnormal renal function	0.91 (0.79 to 1.05)
2 [10,11]	448	Hepatic dysfunction	0.83 (0.64, 1.09)
4 [10,11,21,25]	526	Sepsis	1.04 (0.45 to 2.39)
5 [10,11,21,24,25]	545	Seizures	1.04 (0.91 to 1.18)
2 [10,25]	292	Hypokalemia	1.02 (0.84 to 1.25)

## Discussion

Our results suggest that in general, cooling of neonates with HIE has a beneficial effect on the primary composite outcome of death or disability at 18–22 months. The similarity of outcomes between trials despite the heterogeneity related to various factors including methodology of cooling (head vs. body, devices, target temperatures, site of monitoring, duration of intervention etc.), patient characteristics (place of birth, temperature and age at enrolment etc.), and the definition and degree (moderate and/or severe) of neuro-developmental disability (Table 1) is reassuring for the generalisability of the findings. Differences in the behavior of the composite outcome vs. its individual components are an important consideration [30]. The selection of death and neurodevelopmental disability as components of the prespecified primary composite outcome is justified. The frequency of the components of the primary composite outcome is significant and comparable, assuring that no individual component is driving it in any specific direction. The data show reasonably convincingly that the benefit for the primary composite outcome is significant and is probably a fair reflection of benefit for its individual components. Individual components of the primary composite outcome show significant benefits of cooling but have wide CIs suggesting that more data is needed to minimize the uncertainty. However, it is important to note that interpretation of CI is a personal and subjective issue. Overall, there seems to be reasonably good evidence of a real benefit of cooling on the primary composite outcome and its components.

The definition of disability in the whole body cooling trial by Shankaran *et al *is quite different from that in the selective head cooling trials by Gluckman *et al *and Gunn *et al *(*moderate/severe vs. only severe disability*). However the effect of this difference in definitions on the results of the meta-analysis is limited because the proportion of infants with moderate disability is low in all trials. Subgroup analysis seems to suggest that the results of the selective head cooling trials are not as convincing as those of the whole body cooling trials. However, there might be no clinically important difference between these cooling techniques for several reasons: Firstly, this subgroup analysis is clearly dominated by the two major trials Gluckman *et al *and Shankaran *et al*, and therefore of limited value. The overall trend for both cooling techniques is towards a benefit (Figure 1). Secondly, differences in the severity of neuronal injury may explain these findings considering the differences in the inclusion criteria of those two trials (Table 2). The proportion of neonates with very low Apgar scores, severe aEEG background activity and severe clinical encephalopathy was higher in the intervention group in Gluckman *et al*, possibly reducing the chances to demonstrate selective head cooling benefits. A significant reduction in death or major disability was however noted in the prespecified subgroup analysis of neonates with only moderate injury defined by aEEG criteria [10,31]. Thirdly, palliation bias in the form of a higher rate of withdrawal of treatment (27 vs. 12) in the control group may also have played a role in the significant benefit reported in the whole body cooling trial by Shankaran *et al *[32]. In addition, 41/106 neonates in the control group of Shankaran *et al*, at least once had a temperature > 38°C within the 72 hours of the intervention, which may have influenced for their outcome [32].

The frequency of sinus bradycardia following therapeutic hypothermia was significant. A borderline effect on thrombocytopenia was noted and there was a trend towards a higher risk of anemia, coagulopathy, and hypotension (Table 5). However, sinus bradycardia is a physiological response rather than a true adverse event and did not compromise perfusion, and thrombocytopenia was not reported as of clinical importance. The adverse events therefore may be outweighed by the potential benefits.

Given the overall encouraging results without significant adverse effects it is not surprising that some centers may now consider therapeutic hypothermia as a standard treatment for HIE [33]. However, many experts including a commission of the American Academy of Pediatrics [34] have suggested that further research should continue and therapeutic hypothermia should not be offered outside RCTs. Their suggestions are based on heterogeneity as discussed above and the possibility that neurological outcomes at 18 to 22 months may not reflect the true long-term benefits [35,36]. The rate of severe disability is very unlikely to change, however, more subtle neurodevelopmental problems that cannot be assessed at the age of 18 months may become apparent by school age [1]. The unaddressed issues include the specific target population that is most likely to benefit, the most effective and safe method for cooling, the optimal age at onset and duration of cooling, and the field difficulties in applying any specific method for cooling, particularly for outborn neonates [34]. In practice, hypothermia is quite frequent in asphyxiated neonates, whereas guidelines for rewarming are not standardised/uniform. The targets, methods as well as the speed of rewarming may influence the neuronal recovery/damage following HIE. This issue is especially important during transport of hypothermic neonates with HIE. The field difficulties have been addressed to some extent by Eicher *et al *who showed that it is feasible to cool outborn neonates with ice bags followed by cooling with a blanket on reaching the receiving hospital. This approach can help to reduce the time between birth asphyxia and initiation of cooling. Animal studies have clearly shown that there is a correlation between early onset of cooling and treatment effect [37].

Experts have advised that centers wishing to offer therapeutic hypothermia outside RCTs should adhere strictly to a trial protocol and have established the substantial resources required to cool neonates with HIE. The minimum resources include a transport team to retrieve neonates and start cooling before four to six hours of life and a multidisciplinary team for long-term neurodevelopmental follow-up [33].

At least three more RCTs of therapeutic hypothermia aiming at a combined total of over 650 neonates are currently in progress [38-40]. The long-term outcomes ≥ 18 months of age of the single trial with complete recruitment (according to the TOBY trial website) will not be ready for publication until end of 2008 at the earliest [38]. Ideally it is preferable to include the long-term results of those ongoing studies in this systematic review to have definitive answers. However, those studies are not designed to answer all the unaddressed issues listed earlier, therefore waiting for their results is ethically complex. They may show beneficial effects of therapeutic hypothermia while narrowing the CI. In that case it is disturbing to think that while waiting for the CI to narrow the purists may have denied a beneficial intervention to neonates with HIE. Obviously ethics [41], resources, parents' wishes, and last but not the least, the anxiety related to future medicolegal challenges, will have to be balanced before deciding whether therapeutic hypothermia can be offered as a standard treatment for HIE. Continuing to participate in a trial of therapeutic hypothermia while offering it to neonates whose parents insist on it probably violates the principle of equipoise, the very justification for conducting a RCT.

## Conclusion

Evidence from high quality RCTs indicates that overall, cooling of neonates with moderate to severe HIE reduces the risk of death or disability at 18 to 22 months without significant adverse effects. Despite the methodological differences, wide CIs, and lack of long-term follow-up data, the consistency of benefits and its sound scientific basis indicate that cooling may be an attractive option for neuroprotection in HIE – a condition that lacks any effective treatment at present.

## Competing interests

The author(s) declare that they have no competing interests.

## Authors' contributions

SKP and SMS designed the review protocol. All authors searched the literature independently and assessed inclusion criteria as well as quality of the trials. SMS and SR independently extracted the data. SMS and SKP wrote the manuscript. All authors read and approved the final manuscript.

## Pre-publication history

The pre-publication history for this paper can be accessed here:


